# 15q26缺失综合征伴纯红细胞再生障碍1例

**DOI:** 10.3760/cma.j.issn.0253-2727.2023.06.017

**Published:** 2023-06

**Authors:** 启国 张, 文静 傅, 文玉 宫, 其川 金

**Affiliations:** 滁州市第一人民医院，安徽医科大学附属滁州医院，滁州 239001 The First People's Hospital of Chuzhou, Chuzhou Hospital Affiliated to Anhui Medical University, Chuzhou 239001, China

患者，女，44岁。主因“反复乏力、晕厥3个月”入院。查体：体重30.0 kg，身高130 cm，发育不良，重度贫血面容，右手拇指畸形，双手部分手指弯曲状。既往史：自幼中耳炎史，后渐耳聋、失聪，未就医诊治。家族史：患者父亲已故；母亲体健，身高约150 cm，怀孕时有多种药物服药史；丈夫身体健康，育一女，身体健康，身高160 cm，智力正常。血常规：WBC 4.05×10^9^/L，HGB 59 g/L，PLT 213×10^9^/L，网织红细胞比值（Ret）0.30％，平均红细胞体积（MCV）94.7 fl，红细胞分布宽度（RDW）13.8％，平均血红蛋白浓度（MCHC）333 g/L。血清铁蛋白590.19 µg/L，叶酸>45.30 nmol/L，维生素B_12_ 414.05 pmol/L。自身抗体全套：抗核抗体（ANA) 核颗粒型（1∶80）阳性，抗心磷脂抗体弱阳性，余正常。细小病毒B19 IgG、IgM均阴性。外周血T淋巴细胞亚群：CD3^+^ 90％，CD3^+^CD4^+^ 60％，CD3^+^CD8^+^ 27％。溶血性贫血组套、直接抗人球蛋白试验（DAT）、CD55、CD59正常。骨髓涂片：增生活跃骨髓象，红系有核红细胞少见，仅晚期幼稚红细胞0.5％。粒系中原始粒细胞以下可见，中、晚期幼稚粒细胞及分叶核粒细胞比例偏高，形态大致正常。全片巨核细胞15个，其中颗粒巨核细胞8个，产板巨核细胞5个，裸核型巨核细胞2个。骨髓铁染色：外铁（++）；内铁：有核红细胞少见，内铁阳性率不宜评估。骨髓活检：未见幼稚细胞增多，巨核细胞未见明显病态造血，红系细胞少见；染色体核型：46，XX[20]。骨髓流式细胞未检测到明显的免疫表型异常证据。血液病常见56种融合基因筛查阴性。骨髓标本ARID1B基因插入突变：c.1041_1043dupGGC（p.A350dupA），突变频率31.9％。丝裂霉素C染色体畸变结果正常。彗星试验：外周血淋巴细胞DNA存在损伤，彗星细胞率17％，未见凋亡细胞。遗传性血液病相关基因单核苷酸变异（SNV）检测：①未检测到受检者血液系统遗传病相关基因的微小致病性基因变异；②拷贝数变异（CNV）检测提示15号染色体q26.2q26.3区域存在大小约5.1 Mb的大片段杂合缺失，该区域包含40个基因，其中OMIM收录的基因有MEF2A、LINS1、ADAMTS17、CERS3、ALDH1A3、CHSY1、IGF1R等，建议应用CMA方法进行确认。染色体微阵列（CMA）检测：应用CytoScan HD芯片证实chr15q26.2q26.3存在约 5.2 Mb缺失，基因组位点：arr [GRCh37]15q26.2q26.3(97236143_102429112)x1，CNV类型：缺失，长度：5.2 Mb，与15q26缺失综合征有关。诊断：15q26 末端缺失综合征、纯红细胞再生障碍（PRCA）。予泼尼松1 mg/kg治疗，3周后复查HGB 90 g/L，WBC 10.80×10^9^/L，PLT 360×10^9^/L，摆脱输血依赖，目前一般情况良好，3个月后血常规完全恢复正常。

讨论：1977年Drayer等报道一独特家庭的2个姐弟，特征是智力迟钝、小头畸形、身材矮小、指骨缺失，当时推测为常染色体隐性遗传综合征（Drayer's syndrome）。30年后现代分子学技术重新评估该家庭病例发现由重现性15q缺失5.8 Mb片段引起。15q26缺失综合征主要表现为生长发育迟缓、身材矮小、小头畸形、先天性心脏异常、智力低下、面容和四肢畸形等，临床罕见易误诊。15q26缺失综合征的大部分表型效应由少数几个关键基因缺失导致，如本例缺失的胰岛素样生长因子1受体（IGF1R）。获得性PRCA的典型特征是骨髓中红细胞生成完全或近乎完全停止，红细胞生成受抑主要涉及免疫机制。本例贫血改善较快，提示15q26末端微缺失对激素临床疗效未产生明显影响。本病例为国际首例成人期确诊的15q26末端缺失综合征伴PRCA。PRCA伴躯体发育异常者注意鉴别诊断要考虑到15q26末端缺失综合征。

